# Light-Induced Dynamic Change of Phytochrome B and Cryptochrome 1 Stabilizes SINATs in *Arabidopsis*

**DOI:** 10.3389/fpls.2021.722733

**Published:** 2021-08-20

**Authors:** Jin Hu, Yinmeng Hu, Mengran Yang, Xiaotong Hu, Xuelu Wang

**Affiliations:** ^1^College of Life Science and Technology, Huazhong Agricultural University, Wuhan, China; ^2^State Key Laboratory of Crop Stress Adaptation and Improvement, Henan University, Kaifeng, China; ^3^State Key Laboratory of Genetic Engineering and Department of Genetics, School of Life Sciences, Fudan University, Shanghai, China

**Keywords:** phytochrome B, cryptochrome 1, light, photoreceptors, SINA of *Arabidopsis thaliana*

## Abstract

Ubiquitin-dependent protein degradation plays an important role in many plant developmental processes. We previously identified a class of SINA RING-type E3 ligases of *Arabidopsis thaliana* (SINATs), whose protein levels decrease in the dark and increase in red and blue light, but the underlying mechanism is unclear. In this study, we created transgenic lines carrying point mutations in *SINAT* genes and photoreceptors-NLS or -NES transgenic plants to investigate the regulatory mechanism of SINAT protein stability. We demonstrated that the degradation of SINATs is self-regulated, and SINATs interact with photoreceptors phytochrome B (phyB) and cryptochrome 1 (CRY1) in the cytoplasm, which leads to the degradation of SINATs in the dark. Furthermore, we observed that the red light-induced subcellular localization change of phyB and blue light-induced the dissociation of CRY1 from SINATs and was the major determinant for the light-promoted SINATs accumulation. Our findings provide a novel mechanism of how the stability and degradation of the E3 ligase SINATs are regulated by an association and dissociation mechanism through the red light-induced subcellular movement of phyB and the blue light-induced dissociation of CRY1 from SINATs.

## Introduction

Unlike animals, plants are sessile and must integrate their responses to multiple environmental stimuli to optimize growth and development. One way for cells to respond rapidly to environmental changes is to adjust protein levels through protein degradation (Xu and Xue, 2019; Doroodian and Hua, 2021). In eukaryotes, this posttranslational regulation is often mediated by the ubiquitin (Ub) 26S proteasome pathway (Stone, 2014; Zhou and Zeng, 2017; Linden and Callis, 2020). In this pathway, proteins are targeted for degradation by ubiquitination *via* E1 (ubiquitin-activating proteins), E2 (ubiquitin-conjugating proteins), and E3 enzymes (ubiquitin ligases; Smalle and Vierstra, 2004; Marshall and Vierstra, 2019). E3 ligases recognize substrates and direct their interaction with E2s, resulting in highly specific protein ubiquitination and degradation (Xie et al., 2002; Kelley, 2018; Ban and Estelle, 2021). The *Arabidopsis thaliana* genome contains more than 1,000 genes encoding E3 ligases (Gagne et al., 2002; Wu et al., 2020; Li, 2021). Although numerous studies have analyzed the degradation of their specific substrates (Xie et al., 2002; Wang et al., 2009, 2013), few have focused on the degradation of E3 ligases themselves.

SINATs (SINA of *A. thaliana*) are a class of RING-type E3 ligases with five members (SINAT1-5; Yang et al., 2017). RING-type E3 ligases typically contain two functional domains: an N-terminal RING domain which binds to E2 to form a proteolytic complex and a C-terminal SINA domain which regulates oligomerization and binding to target proteins (Hu and Fearon, 1999; Polekhina et al., 2002). SINATs participate in stress responses and growth processes (Park et al., 2010; Ning et al., 2011; Xia et al., 2020), but little is known about the regulation of their protein stability. Recently, we found that SINATs control degradation of the brassinosteroid (BR)-activated transcription factor BES1, but BR signaling does not affect the expression of *SINAT* genes or the abundance of SINATs. Notably, SINAT protein levels are regulated by light, and they decrease in the dark and increase under red and blue light (Yang et al., 2017). However, the underlying biochemical mechanism by which light regulates SINAT protein levels is unknown.

Light regulates every aspect of plant growth and development (Podolec and Ulm, 2018), and plants have several classes of photoreceptors, such as phytochromes, cryptochromes (CRYs), and phototropins, that perceive red and/or blue light (Liu et al., 2011; Yin and Ulm, 2017). The red-light receptor phytochrome B (phyB) is one of the most important members of the phytochrome family (Smith, 2000). The phytochromes predominantly localize to the cytoplasm in the dark and translocate to the nucleus upon exposure to red light (Fankhauser and Chen, 2008). The CRYs are flavoproteins that perceive blue light in plants (Keller et al., 2011; Liu et al., 2016). *Arabidopsis* possesses two homologous cryptochromes, CRY1 and CRY2. CRY1 plays a major role in mediating the blue-light inhibition of hypocotyl elongation, whereas CRY2 mainly functions under low intensities of blue light (Lin et al., 1998). *Arabidopsis* CRY1 located in the cytoplasm and the nucleus has separate functions, and petiole elongation inhibition and anthocyanin production were related to nuclear CRY1 while primary root growth and cotyledon expansion in blue light are promoted by cytoplasmic CRY1 and inhibited by nuclear CRY1, whereas CRY2 is located exclusively in the nucleus (Wu and Spalding, 2007). Although red and blue light induce accumulation of SINATs, it is unknown whether this occurs *via* phyB and/or CRY1.

In this study, we introduced point mutations into the RING domain of SINATs and showed that SINAT stability is regulated *in planta* through an autocatalytic degradation mechanism. We demonstrated that phyB and CRY1 directly interacted with SINATs and promoted SINAT degradation. The photoreceptor/SINAT interactions occurred in the cytoplasm and induced SINAT degradation in the dark. In the red light, translocation of phyB from the cytoplasm to the nucleus caused its dissociation from the SINATs, and the blue light inhibits the interaction between SINATs and CRY1, all leading to the reduced degradation of SINATs. Our study disclosed the cellular and molecular mechanisms of the autocatalytic degradation of SINATs in the dark and the light-promoted protein accumulation of this class of E3 ligases through dissociation from the photoreceptors.

## Materials and Methods

### Plant Materials and Growth Conditions

*Arabidopsis* ecotype Columbia (Col-0) was used as the wild-type control and *SINAT2-FLAG* and *SINAT2^C63S^-FLAG* lines were generated by floral dipping in the Col-0 background. *SINAT2-FLAG phyB-GFP*, *SINAT2-FLAG CRY1-Myc*, *SINAT2-FLAG phyB*, and *cry1 SINAT2-FLAG* lines were obtained by genetic crossing. *Arabidopsis* ecotype Landsberg (Ler) was the wild type. Surface-sterilized seeds were sown on half-strength Murashige and Skoog (MS; pH 5.7–5.9) medium (Phytotechnology) with 0.8% phytagel (Sigma-Aldrich) before being transferred to soil (nutrient soil:roseite=3:1). Plants were grown at 22°C in long-day conditions with a 16-h light/8-h dark cycle.

### Hypocotyl Assay

Seeds were surface-sterilized in 75% ethanol for 10min, followed by absolute ethanol for 10min. After allowing the ethanol to evaporate, the sterile seeds were sprinkled into half-strength MS medium (pH 5.7–5.9) with 0.8% phytagel and stratified in the dark at 4°C for 72h. For *SINAT2-FLAG* and *SINAT2^C63S^-FLAG*, the plants were then exposed to constant white light (20μmolm^−2^ s^−1^) for 7days, for *phyB-N-GFP SINAT2-FLAG phyB*, *phyB-N-GFP-NLS SINAT2-FLAG phyB*, and *phyB-N-GFP-NES SINAT2-FLAG phyB*, the plants were then exposed to constant red light (20μmolm^−2^ s^−1^) for 5days. Individual seedlings were transferred to a transparent background for scanning and measurement. Hypocotyl length was measured using ImageJ software.^1^

### *In vitro* Pull-Down Assay

The coding sequence of phyB-N, phyB-C, CRY1-N, and CRY1-C was cloned into pGEX-4T-1 to obtain GST (Glutathione-S-transferase) recombinant proteins. phyB-C-GST, CRY1-N-GST, CRY1-C-GST, GST, SINAT2-His, and SINAT5-His recombinant proteins were produced in *Escherichia coli* BL21 (DE3) pLysS (TIANGEN). The GST-tagged proteins were purified with glutathione resin (GenScript), and His-tagged proteins were purified with TALON Metal Affinity Resin (Clontech). For pull-down assays, about 5μg of GST-tagged protein was incubated with glutathione resin (GenScript) at 4°C for 1h. After washing with phosphate-buffered saline (PBS), about 2μg of His-tagged protein was added. After incubation for 1h at 4°C, the resin was washed several times with wash buffer (1× PBS, 0.1% Triton X-100) and was then boiled in SDS loading buffer at 95°C for 10min and loaded into SDS polyacrylamide gels, which were subjected to immunoblotting. Anti-His antibody (Abbkine) was used to detect His-tagged proteins and anti-GST antibody (NewEast) was used to detect GST-tagged proteins.

### Semi-*in vivo* Pull-Down Assay

*SINAT2-FLAG* and *SINAT5-FLAG* lines were grown on half-strength MS medium in the incubator (Percival) at 22°C in long-day conditions with a 16-h light/8-h dark cycle for 2weeks. Plant tissues were harvested and total protein was extracted in extraction buffer (100mM Tris-HCl pH 7.5, 300mM NaCl, 2mM EDTA pH 8.0, 1% Triton X-100, 10% glycerol, 50μM MG132, and protease inhibitor 1:100). The extracts were centrifuged twice at 12,000rpm for 10min and the supernatants were incubated with either GST or phyB-N-GST, phyB-C-GST, CRY1-N-GST, or CRY1-C-GST, which had been pre-incubated with GST beads at 4°C for 1h. The beads were washed 4–8 times with wash buffer (1× PBS, 0.1% Triton X-100) and boiled with 1× SDS loading buffer, and the extracted proteins were then separated by SDS-PAGE and immunoblotted with anti-FLAG antibodies (Abmart).

### Co-immunoprecipitation Assay

Overexpression transgenic plants were grown on half-strength MS medium in the incubator (Percival) at 22°C in long-day conditions with a 16-h light/8-h dark cycle for 2weeks. Proteins were extracted with protein extraction buffer (100mM Tris-HCl pH 7.5, 300mM NaCl, 2mM EDTA pH 8.0, 1% Triton X-100, 10% glycerol, 50μM MG132, and protease inhibitor). The extracts were centrifuged twice at 12,000rpm for 10min and the supernatants were collected. Anti-GFP gel (KT health) was used to pull down phyB-GFP from *SINAT2-FLAG phyB-GFP* and *SINAT5-FLAG phyB-GFP*, and anti-FLAG M2 agarose gel (Sigma) was used to pull down SINAT2-FLAG from *SINAT2-FLAG CRY1-Myc* and *SINAT5-FLAG CRY1-Myc*. The beads were washed 3–6 times with wash buffer (1× PBS, 0.1% Triton X-100) and boiled with 1× SDS loading buffer, and the extracted proteins were then separated by SDS-PAGE and immunoblotted with anti-FLAG (Abmart) and anti-Myc (Abmart).

### BiFC Assays and Subcellular Localization Analysis

For bimolecular fluorescence complementation (BiFC) assays, full-length SINAT, SINAT-N, and SINAT-C were cloned into pXY106-nYFP to fuse to N-terminal YFP, and phyB and CRY1 were cloned into pXY104-cYFP to fuse to C-terminal YFP. To detect subcellular localization, the C-termini of SINATs were inserted into the vector *pCAMBIA2300* to create a fusion to GFP, and phyB and CRY1 were inserted into *pCAMBIA1300* to fuse them to mCherry. The resulting vectors and control vectors were introduced into *Agrobacterium tumefaciens* strain GV3101 and then infiltrated into *Nicotiana benthamiana* leaves. YFP fluorescence and subcellular localization were detected using a Leica SP8 confocal microscope.

### Semi-*in vivo* Ubiquitination Assay

The coding sequence of SINAT2 was cloned into PET-28a to obtain His recombinant proteins. The recombinant His-SINAT2 protein bound to TALON Metal Affinity Resin (Clontech) was incubated with equal amounts of protein extracts of *SINAT2-FLAG* and *SINAT2-FLAG phyB* plants in degradation buffer (100mM Tris-HCl pH 7.5, 300mM NaCl, 2mM EDTA pH 8.0, 1% Triton X-100, 10% glycerol, 50mM MG132, and protease inhibitor). After incubated at 30°C for the 2h, The resin was washed three times with the extraction buffer (50mM Tris-HCl pH 7.5, 150mM NaCl, 1mM EDTA pH 8.0, 0.5% Triton X-100, and 5% glycerol) and boiled with SDS loading buffer, separated by SDS-PAGE, and immunoblotted with anti-His antibodies and anti-Ub (SANTA CRUZ BIOTECHNOLOGY) antibodies.

### Construction of Transgenic Lines

To create the transgenic lines *phyB-N-GFP SINAT2-FLAG phyB*, *phyB-N-GFP-NLS SINAT2-FLAG phyB*, *phyB-N-GFP-NES SINAT2-FLAG phyB*, *CRY1-N-GFP cry1 SINAT2-FLAG*, *CRY1-N-GFP-NLS cry1 SINAT2-FLAG*, and *CRY1-N-GFP-NES cry1 SINAT2-FLAG*, the N-termini of phyB and CRY1 were fused with nuclear localization signal (NLS) sequence or nuclear export signal (NES) sequence and cloned into the vector pCAMBIA2300 fused with a GFP tag. The plasmids were transferred into *SINAT2-FLAG phyB* and *cry1 SINAT2-FLAG via* Agrobacterium strain GV3101.

## Results

### SINAT Protein Degradation in the Dark Is Self-Regulated

**Figure 1:**
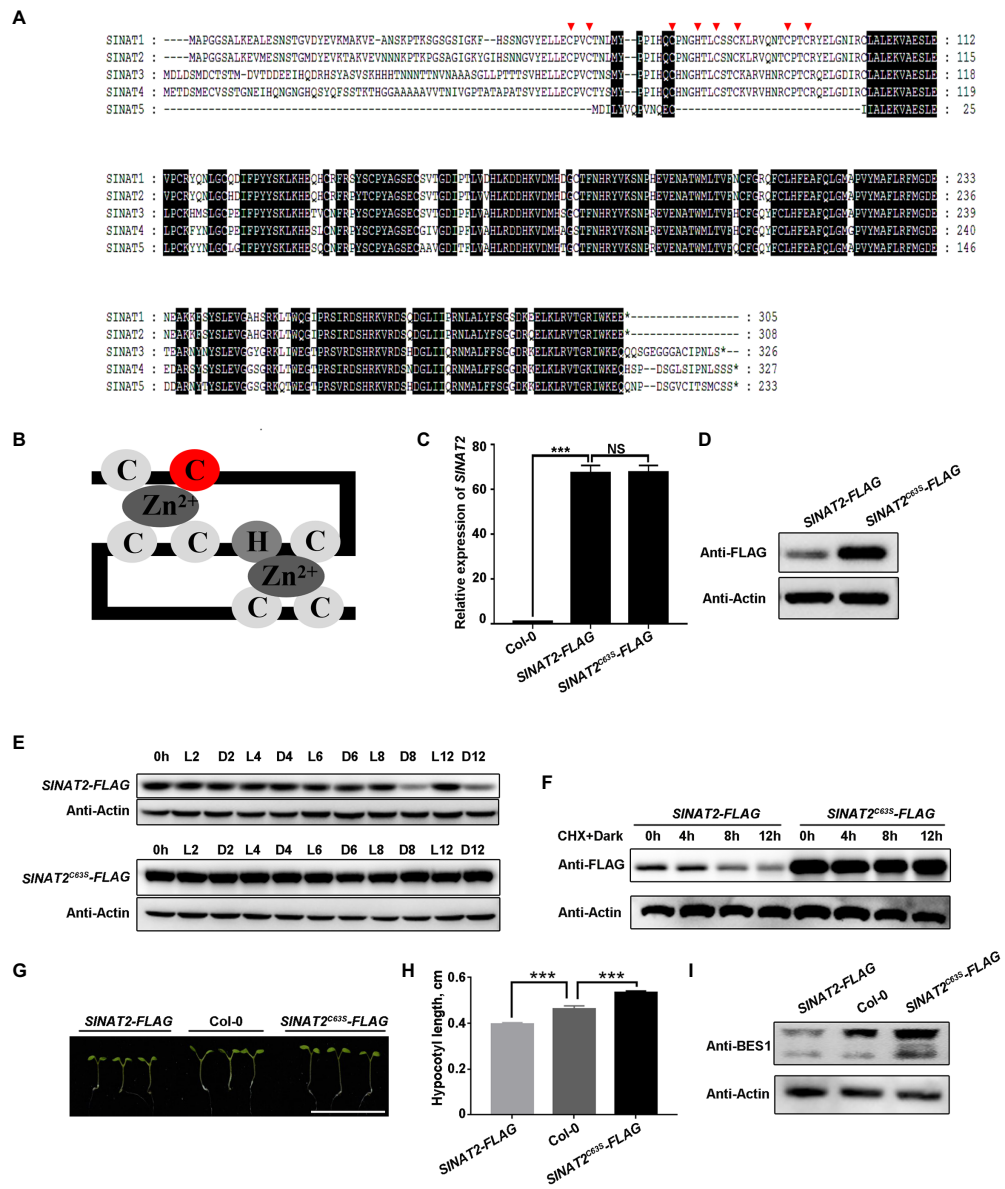
SINAT protein degradation in the dark is self-regulated. **(A)** Protein sequence of five SINATs in *Arabidopsis thaliana*: SINAT1 (At2g41980), SINAT2 (At3g58040), SINAT3 (At3g61790), SINAT4 (At4g27880), and SINAT5 (At5g53360). The conserved regions are indicated with a black background. The red arrows mark the eight conserved residues in the RING domain. **(B)** Diagram of how the eight conserved amino acids form a zinc finger; the second cysteine residue (C63 in SINAT2; C67 in SINAT4) is marked in red. **(C)** *SINAT2* transcript levels in Col-0 and the *SINAT2-FLAG* and *SINAT2^C63S^-FLAG* transgenic lines. Data are means±SD (*n*=3) and values of *p* were determined by Student’s *t*-test; ^***^p<0.001; NS, non-significant, *p*>0.05. **(D)** SINAT2 protein levels in the *SINAT2-FLAG* and *SINAT2^C63S^-FLAG* transgenic lines. An anti-FLAG antibody was used to detect SINAT2 and an anti-Actin antibody was used to quantify the total protein. **(E)** SINAT2 protein levels under different light conditions. The *SINAT2-FLAG* and *SINAT2^C63S^-FLAG* transgenic lines were grown in continuous light (L) for 5days and were then kept in the light or transferred to the dark (D) for 2, 4, 6, 8, or 12h. **(F)** The half-life of SINAT2 protein in the *SINAT2-FLAG* and *SINAT2^C63S^-FLAG* lines. These lines were grown in continuous light (L) for 5days and were then treated with cycloheximide (CHX) and transferred to the dark (D) for 4, 8, or 12h. **(G)** Hypocotyl phenotypes of Col-0, *SINAT2-FLAG*, and *SINAT2^C63S^-FLAG* lines. The scale bar corresponds to 1cm. **(H)** Hypocotyl length of Col-0 and the *SINAT2-FLAG* and *SINAT2^C63S^-FLAG* lines. Error bars indicate the SE (*n*=30). Statistical significance was determined by Student’s *t*-test. ^***^p<0.001. **(I)** BES1 protein levels in Col-0 and the *SINAT2-FLAG* and *SINAT2^C63S^-FLAG* lines. Anti-BES1 antibody was used to detect BES1 protein; anti-Actin antibody was used to quantify the total protein.

To investigate whether SINAT degradation is self-regulated, we analyzed the amino acid sequences of the proteins encoded by the five *SINAT* genes (*SINAT1*, *At2g41980*; *SINAT2*, *At3g58040*; *SINAT3*, *At3g61790*; *SINAT4*, *At4g27880*; and *SINAT5*, *At5g53360*) and found that all except SINAT5 contain a RING domain. The RING domain is essential for the enzymatic function of the SINATs and contains eight conserved amino acid residues (Figure 1A) that chelate two Zn ions to form a zinc-finger structure (Figure 1B; Chen et al., 2002; Yang et al., 2017). We created a SINAT2^C63S^ mutant with a mutation in the second cysteine residue of the RING domain’s eight conservative residues and generated transgenic plants that overexpressed *SINAT2^C63S^* and wild-type *SINAT2* in Col-0. We then compared the SINAT2 protein levels in lines with similar levels of *SINAT2* or *SINAT2^C63S^* transcripts and observed that SINAT2^C63S^ accumulated more than wild-type SINAT2 (Figures 1C,D; Supplementary Figures 1A,B). We also tested the stability of SINAT2^C63S^ and wild-type SINAT2 in the transgenic lines under changing light conditions. When plants were transferred from the light to the dark, the level of wild-type SINAT2 decreased greatly, as reported previously (Yang et al., 2017; Yang and Wang, 2017), but the level of SINAT2^C63S^ remained almost unchanged (Figure 1E; Supplementary Figure 1C), indicating that SINAT2^C63S^ was more stable than SINAT2 in the dark. We measured the half-life of SINAT2-FLAG and SINAT2^C63S^-FLAG in plants treated with cycloheximide (CHX) in the dark and observed that SINAT2-FLAG was degraded much faster than SINAT2^C63S^-FLAG (Figure 1F; Supplementary Figure 1D). Collectively, these results suggest that the degradation of SINATs in the dark probably requires their own E3 ligase activity.

To investigate the effect of SINAT2^C63S^ on plant growth, we germinated *SINAT2-FLAG* and the *SINAT2^C63S^-FLAG* plants under continuous dim light and measured their hypocotyl lengths. The hypocotyls of the *SINAT2-FLAG* plants were shorter than those of wild-type Col-0, but hypocotyls of *SINAT2^C63S^-FLAG* plants were significantly longer (Figures 1G,H; Supplementary Figures 1E,F). We also quantified endogenous BES1, a known substrate of SINATs, in the transgenic lines (Yang et al., 2017; Yang and Wang, 2017). The level of BES1 protein was higher in the *SINAT2^C63S^-FLAG* plants than in Col-0 or the *SINAT2-FLAG* plants (Figure 1I; Supplementary Figure 1G), indicating that the *SINAT2^C63S^* lost its E3 ligase activity, leading to BES1 protein accumulation and seedling hypocotyl elongation.

### SINATs Interact With phyB and CRY1

**Figure 2:**
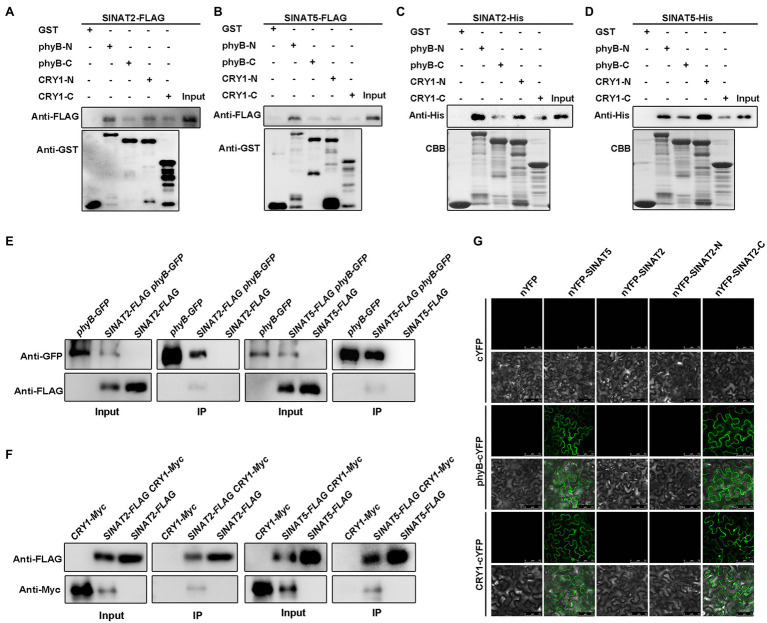
SINATs interact with phyB and CRY1. **(A)** Interaction of SINAT2 with phyB-N/phyB-C and CRY1-N/CRY1-C by semi-*in vivo* pull-down assays. **(B)** Interaction between SINAT5 and phyB-N/phyB-C or CRY1-N/CRY1-C detected by semi-*in vivo* pull-down assays. **(C)** Interaction between SINAT2 and phyB-N/phyB-C or CRY1-N/CRY1-C detected by *in vitro* pull-down assays. Approximately equal loading of the recombinant proteins was determined by Coomassie Brilliant Blue (CBB) staining. **(D)** Interaction between SINAT5 and phyB-N/phyB-C or CRY1-N/CRY1-C detected by *in vitro* pull-down assays. **(E)** Interaction between SINAT2 or SINAT5 and phyB detected by co-immunoprecipitation (Co-IP). The phyB-GFP was immunoprecipitated with anti-GFP gel, and the co-immunoprecipitated SINAT2-FLAG or SINAT5-FLAG was detected by Western blotting (WB) with anti-FLAG antibodies. **(F)** Interaction between SINAT2 or SINAT5 and CRY1 detected by Co-IP. The SINAT2-FLAG or SINAT5-FLAG was immunoprecipitated with anti-FLAG agarose, and the co-immunoprecipitated CRY1-Myc was detected by Western blotting (WB) with anti-Myc antibodies. **(G)** Interaction between SINAT2 and SINAT5 with phyB and CRY1 in bimolecular fluorescence complementation (BiFC) assays. The indicated constructs were transformed into pavement cells of *Nicotiana benthamiana*.

To study how light promotes SINATs accumulation, we tested whether the SINATs could interact with the red-light receptor phyB and the blue-light receptor CRY1. Because it is difficult to express the full-length phyB and CRY1 proteins, we used truncated recombinant phyB proteins that included either the N-terminus (phyB-N, amino acids 1–645) or the C-terminus (phyB-C, amino acids 646–1,172) and truncated recombinant CRY1 proteins that included either the N-terminus (CRY1-N, amino acids 1–490) or the C-terminus (CRY1-C, amino acids 491–681; Supplementary Figure 2A), which were separately fused to a GST tag and purified from *E. coli*. Semi-*in vivo* pull-down assays with *SINAT2-FLAG* and *SINAT5-FLAG* transgenic seedlings showed that phyB-N-GST, phyB-C-GST, CRY1-N-GST, and CRY1-C-GST could pull down SINAT2-FLAG or SINAT5-FLAG (Figures 2A,B). The purified SINAT2-His and SINAT5-His proteins from *E. coli* could directly interact with phyB-N-GST, phyB-C-GST, CRY1-N-GST, and CRY1-C-GST *in vitro* (Figures 2C,D). Furthermore, analysis of transgenic plants containing *phyB-GFP* or *CRY1-Myc* and *SINAT2-FLAG* or *SINAT5-FLAG* showed that SINAT2-FLAG and SINAT5-FLAG could co-immunoprecipitate phyB-GFP (Figure 2E) and CRY1-Myc (Figure 2F). However, BiFC assays using full-length SINATs and phyB or CRY1 only showed an interaction between SINAT5 and phyB/CRY1 (Figure 2G). Because SINAT5 lacks a RING domain, we hypothesized that the presence of the RING domain in SINATs causes the degradation of SINATs. To test this, we fused the N-terminal RING domain (SINAT-N) and the C-terminal SINA domain (SINAT-C) of SINAT1-4 (Supplementary Figure 2B) with the YFP N-terminus (nYFP) and conducted BiFC assays in *N. benthamiana* pavement cells. We observed an interaction between SINAT2-C and phyB/CRY1 (Figure 2G) and also between SINAT1-C, SINAT3-C, and SINAT4-C and phyB/CRY1 (Supplementary Figure 3). Taken together, these data suggest that the SINATs interact with phyB/CRY1 *in vitro* and *in vivo* and that these interactions might promote the degradation of the full-length SINATs.

### phyB and CRY1 Promote Degradation of the SINATs

**Figure 3:**
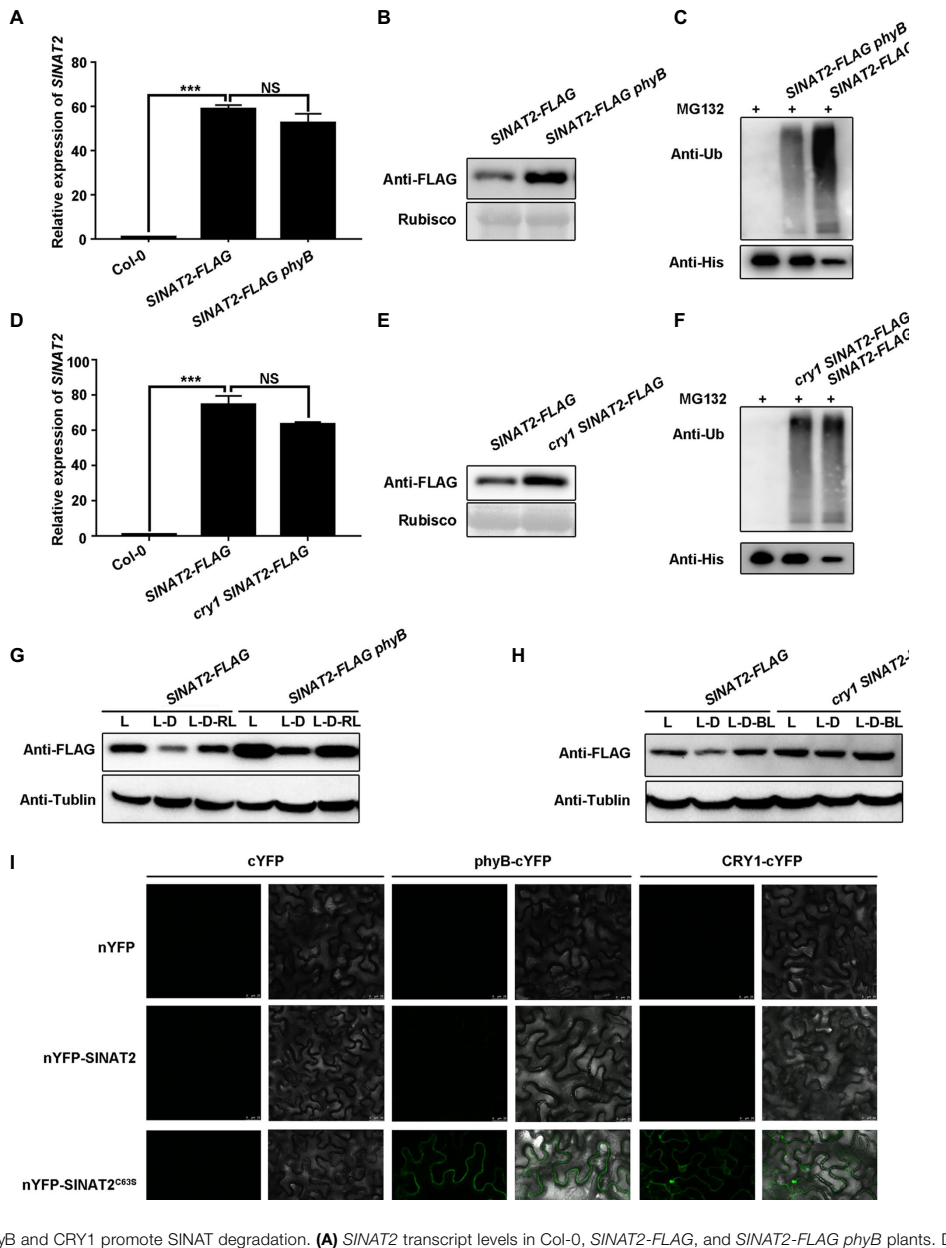
phyB and CRY1 promote SINAT degradation. **(A)** *SINAT2* transcript levels in Col-0, *SINAT2-FLAG*, and *SINAT2-FLAG phyB* plants. Data are means±SD (*n*=3) and values of *p* were determined by Student’s *t*-test; ^***^p<0.001; NS, non-significant, *p*>0.05. **(B)** SINAT2 protein levels in *SINAT2-FLAG* and *SINAT2-FLAG phyB* plants. Rubisco was used as a loading control. **(C)** Detection of His-SIANT2 ubiquitination in the semi-*in vivo* ubiquitination assays. Recombinant His-SIANT2 protein was incubated with protein extracts from the *SINAT2-FLAG* and *SINAT2-FLAG phyB* plants. His-SIANT2 and His-SIANT2-Ub proteins were detected by Western blotting with anti-Ub antibodies (upper gels) and anti-His antibodies (lower gels). **(D)** *SINAT2* transcript levels in Col-0, *SINAT2-FLAG*, and *cry1 SINAT2-FLAG* plants. Data are means±SD (*n*=3) and values of *p* were determined by Student’s *t*-test; ^***^p<0.001; NS, non-significant, *p*>0.05. **(E)** SINAT2 protein levels in *SINAT2-FLAG* and *cry1 SINAT2-FLAG* plants. **(F)** Detection of His-SIANT2 ubiquitination in the *SINAT2-FLAG* and *cry1 SINAT2-FLAG* plants. **(G)** The effect of red light on SINAT2 protein levels in *SINAT2-FLAG* and *phyB SINAT2-FLAG* plants. Five-day-old plants grown under continuous light (L) were transferred to the dark for 6h (L-D) and were then transferred to red light for a further 6h (L-D-RL). **(H)** The effect of blue light on SINAT2 protein levels in *SINAT2-FLAG* and *cry1 SINAT2-FLAG* plants. Five-day-old plants grown under continuous light (L) were transferred to the dark for 6h (L-D) and were then transferred to blue light for a further 6h (L-D-BL). **(I)** Interaction of SINAT2^C63S^ with phyB and CRY1 in BiFC assays.

To test whether phyB and CRY1 could cause degradation of the SINATs, we created *SINAT2-FLAG phyB* (Supplementary Figure 4A) and *cry1 SINAT2-FLAG* lines by crossing the *SINAT2-FLAG* transgenic plants with a *phyB* mutant (*phyB-9*) or *cry1* mutant. Lines with a similar expression level of *SINAT2* were selected for its protein level comparison (Figures 3A,D; Supplementary Figures 5A, C). SINAT2 protein was accumulated to a greater level in *SINAT2-FLAG phyB* and *cry1 SINAT2-FLAG* plants than in *SINAT2-FLAG* plants (Figures 3B,E; Supplementary Figures 5B,D). The 5-day-old, light-grown seedlings were transferred to the dark (L-D) for 6h and then transferred to red or blue light (L-D-RL/L-D-BL) for 6h. SINAT2 was more stable in *SINAT2-FLAG phyB* and *cry1 SINAT2-FLAG* plants than in *SINAT2-FLAG* plants (Figures 3G,H). Furthermore, semi-*in vivo* ubiquitination assays using *SINAT2-FLAG* plants and *SINAT2-FLAG phyB*/*cry1 SINAT2-FLAG* showed that loss of phyB/CRY1 reduced the ubiquitination of SINAT2 (Figures 3C,F). BiFC assays detected interaction between phyB/CRY1 and SINAT2^C63S^ and SINAT4^C67S^ (the SINAT4 mutant with a mutation in the second cysteine residue of the RING domain’s eight conservative residues; Figure 3I; Supplementary Figure 6). Therefore, the physical interaction between SINATs and phyB/CRY1 promoted degradation of the SINATs.

### Red Light-Induced Nuclear Movement of the phyB and Blue Light-Induced Dissociation of CRY1 From SINATs Stabilize SINAT Protein

We previously demonstrated that red and blue light promote SINAT protein accumulation (Yang et al., 2017) but interestingly here show that direct physical interaction between the phyB and CRY1 photoreceptors and SINATs induced degradation of the latter. It was reported that red light promotes phyB to undergo Pr to Pfr change and form homodimer to enter the nucleus, and blue light activates CRY1 to undergo conformational change in both cytoplasm and nucleus to regulate different physiological processes (Klose and Venezia, 2015; Xu et al., 2018; Shao et al., 2020). Therefore, we hypothesized that the light-regulated changes of the photoreceptors may alter their ability to physically contact SINATs.

**Figure 4:**
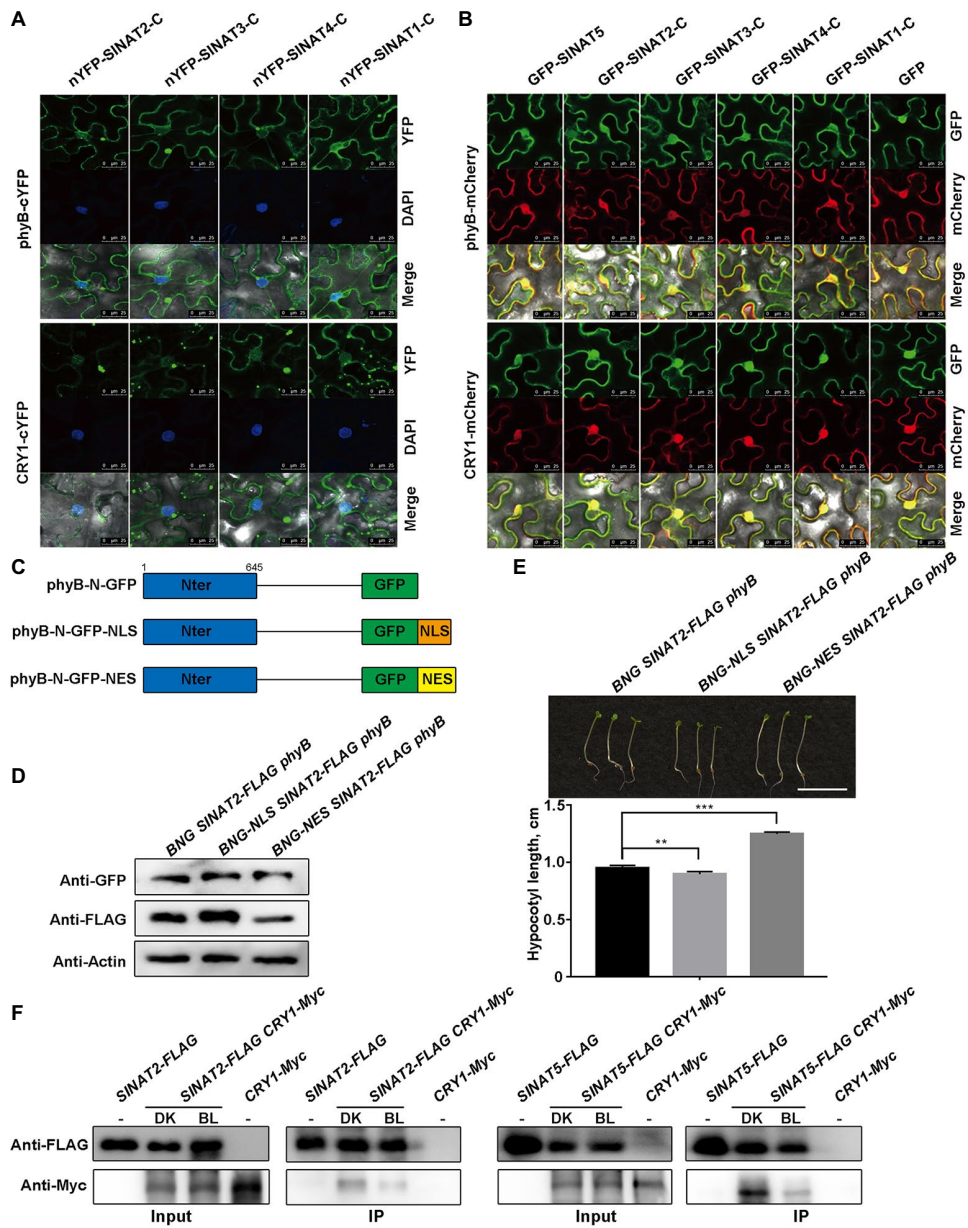
Red light-induced nuclear movement of the phyB and blue light-induced dissociation of CRY1 from SINATs stabilize SINAT protein. **(A)** The C-terminal SINA domain of SINATs interacts with phyB and CRY1 in the cytoplasm. DAPI was used to stain the nucleus. **(B)** Co-localization of SINAT-C proteins with phyB-mCherry/CRY1-mCherry in the cytoplasm and the nucleus. **(C)** Diagrams of the phyB variants. phyB-N was fused to a GFP tag. **(D)** SINAT2 protein levels in *BNG SINAT2-FLAG phyB*, *BNG-NLS SINAT2-FLAG phyB*, and *BNG-NES SINAT2-FLAG phyB* transgenic plants. An anti-GFP antibody was used to detect the phyB-GFP fusion proteins. An anti-FLAG antibody was used to detect SINAT2-FLAG. **(E)** Hypocotyl phenotype and length of *BNG SINAT2-FLAG phyB*, *BNG-NLS SINAT2-FLAG phyB*, and *BNG-NES SINAT2-FLAG phyB* lines. Error bars indicate the SE (*n*=30). Statistical significance was determined by Student’s *t*-test. ^**^p<0.01 and ^***^p<0.001. **(F)** Co-IP assays showing blue light promote the dissociation of CRY1 from SINATs.

To test where the interactions between the C-termini of SINATs (SINATs-C) and phyB/CRY1 occur within the cell, we conducted BiFC assays and found that they mainly interacted in the cytoplasm (Figure 4A). At the same time, we observed the subcellular localization of GFP-SINATs-C, GFP-SINAT5, and phyB-mCherry/CRY1-mCherry and found that all of them were localized to both nucleus and cytoplasm (Figure 4B). Therefore, it was suggested that although phyB/CRY1 can be localized in both cytoplasm and nucleus, SINATs and phyB/CRY1 mainly interact in cytoplasm.

To determine whether the differential subcellular localization of the phyB plays important role in SINAT accumulation, a NLS or a NES was fused to the N-termini of phyB (Figure 4C). In the *N. benthamiana* pavement cells, the transiently expressed phyB-N-GFP (BNG) was located in the nucleus and the cytoplasm, whereas the phyB-N-GFP-NLS (BNG-NLS) was located only in the nucleus, and phyB-N-GFP-NES (BNG-NES) was present only in the cytoplasm (Supplementary Figure 7). We then generated transgenic lines that expressed *phyB-N-GFP (BNG)*, *phyB-N-GFP-NLS (BNG-NLS)*, or *phyB-N-GFP-NES (BNG-NES)* in the *SINAT2-FLAG phyB* background to determine whether SINAT2 protein stability was affected by the localization of phyB. We measured the SINAT2 protein level in the transgenic lines that expressed a considerable amount of the phyB-N protein and observed that more SINAT2 accumulated in *BNG-NLS SINAT2-FLAG phyB* plants than in *BNG SINAT2-FLAG phyB* plants, but less SINAT2 accumulated in *BNG-NES SINAT2-FLAG phyB* plants than in *BNG SINAT2-FLAG phyB* plants (Figure 4D; Supplementary Figure 8A). Then, we germinated those plants under continuous red light and measured their hypocotyl length. The hypocotyls of the *BNG-NLS SINAT2-FLAG phyB* plants were shorter than that of the *BNG SINAT2-FLAG phyB*, but the hypocotyls of the *BNG-NES SINAT2-FLAG phyB* plants were significantly longer than that of the *BNG SINAT2-FLAG phyB* (Figure 4E; Supplementary Figure 8B). At the same time, we detect the ubiquitination of SINAT2 in *BNG SINAT2-FLAG phyB* and *BNG-NLS SINAT2-FLAG phyB* plants and found the ubiquitination of SINAT2 was stronger in *BNG SINAT2-FLAG phyB* than in *BNG-NLS SINAT2-FLAG phyB* (Supplementary Figure 8C), and SINAT2 degrades obviously in *BNG-NES SINAT2-FLAG phyB* without MG132 (Supplementary Figure 8D). All these data indicated that red light-induced alteration in the subcellular localization of phyB led to SINAT protein accumulation.

Although it is unknown whether blue light regulates the nuclear and cytoplasmic migration of CRY1, it is possible that the blue light-activated CRY1 could dissociate from SINATs. In order to verify this hypothesis, we conducted Co-IP assays using overexpressing transgenic plants *SINAT2-FLAG CRY1-Myc* or *SINAT5-FLAG CRY1-Myc*, they were grown for 2weeks and then exposed to dark (DK) or blue light (BL) for 6h. The results showed that the interaction between CRY1 and SINATs is stronger in the dark than that in the blue light (Figure 4F). Taken together, the results indicate that red light-induced alterations in the subcellular localization of phyB and blue light-activated CRY1 cause their dissociation from SINATs, which leads to SINAT protein accumulation in red and blue light.

## Discussion

Our current and previous studies provide several lines of evidence supporting that SINAT degradation is autocatalytic. First, the mutated SINAT2^C63S^-FLAG protein was more stable than the SINAT2-FLAG in plants. Furthermore, when plants were transferred from the light to the dark, the SINAT2^C63S^ protein was also more stable than the wild-type SINATs, and the half-life of the SINAT2^C63S^ protein was significantly longer than the wild-type protein. In addition, the self-ubiquitination of SINAT1-4 and SINAT5^Ler^ was observed in our previous study (Yang et al., 2017). SINATs function in a variety of processes in plants, including hypocotyl elongation, lateral root development, responses to nutrient deficiency and drought, modulates abscisic acid signaling, and plant immunity (Ning et al., 2011; Nolan et al., 2017; Qi et al., 2017; Xia et al., 2020; Xiao et al., 2020). Autocatalytic degradation of E3 ligases is important for regulators involved in diverse biological processes. Previous studies demonstrated that point mutations within BCA2 (a RING-type E3 ubiquitin ligase) in breast tumors negatively affect BCA2 autoubiquitination and result in the accumulation of the BCA2 protein to affect cell migration in mammalian systems (Amemiya et al., 2008). In *Arabidopsis*, a mutant form of the E3 ligase COP1 (Constitutive Photomorphogenic 1) with a defect in the RING domain was refractory to ATM (ataxia telangiectasia mutated)-induced degradation in response to DNA damage (Dornan et al., 2006). At the same time, SINAT proteins were reported to function as oligomers *in vivo* (Xia et al., 2020). Here, overexpression of the mutated *SINAT2^C63S^*, which lacks E3 ligase activity and does not significantly affect the stability of phyB protein (Supplementary Figure 4B), led to seedling hypocotyl elongation.

Light regulates the SINAT protein stability by their physical interaction with the photoreceptors phyB/CRY1. First, phyB and CRY1 interact with SINATs *in vivo* and *in vitro*. Second, SINAT2 accumulated to a greater extent in *SINAT2-FLAG phyB* and *cry1 SINAT2-FLAG* plants than in *SINAT2-FLAG* plants. Third, the semi-*in vivo* system also found that phyB and CRY1 enhanced ubiquitination of the SINATs. Phytochrome is a dimeric chromoprotein (Smith, 2000; Matsushita et al., 2003), and the CRY monomer undergoes a conformational change during activation to form active dimers or oligomers (Kasahara et al., 2002; Shao et al., 2020). Apparently, the phyB/CRY1 proteins function as regulators to affect the auto-degradation of the SINATs. Because the SINATs are involved in many developmental processes by regulating the ubiquitination and degradation of specific substrates (Zhang et al., 2019), and the phyB/CRY1 participates in almost every aspect of plant growth and development, their interaction and regulation provide novel examples for plants integrating environmental cues to regulate plant development.

Apparently, the light-promoted nuclear localization of phyB and blue light-induced dissociation of CRY1 from SINATs enhances the accumulation of SINATs in the light. First, our BiFC assays detected a physical interaction between phyB/CRY1 and SINATs-C in the cytoplasm but not full-length SINATs or SINATs-N, indicating that the latter two proteins formed unstable interactions with phyB/CRY1 in the cytoplasm. Furthermore, in the transgenic plants containing the phyB fused with NLS, the SINAT2 protein was accumulated, but in the transgenic plants containing phyB fused with NES, the level of SINAT2 protein was lower than that in wild-type plants, at the same time, we found the interaction between SINATs and CRY1 was weaker under the control of blue light, indicating that the nuclear-localized phyB and light-induced CRY1 will dissociate from the SINATs and lead to the reduced degradation of SINATs in the light. Differently, light receptor phyB can stimulate EIN3 degradation in the nucleus by recruiting EIN3 to the SCF ubiquitin E3 ligases EBF1/EBF2, which leads to rapid light-induced de-etiolation after seedling emergence from the soil (Shi et al., 2016). The distinct mechanism by which the photoreceptors regulate the SINAT stability provides another layer of protein level control for different substrates and biological processes.

**Figure 5:**
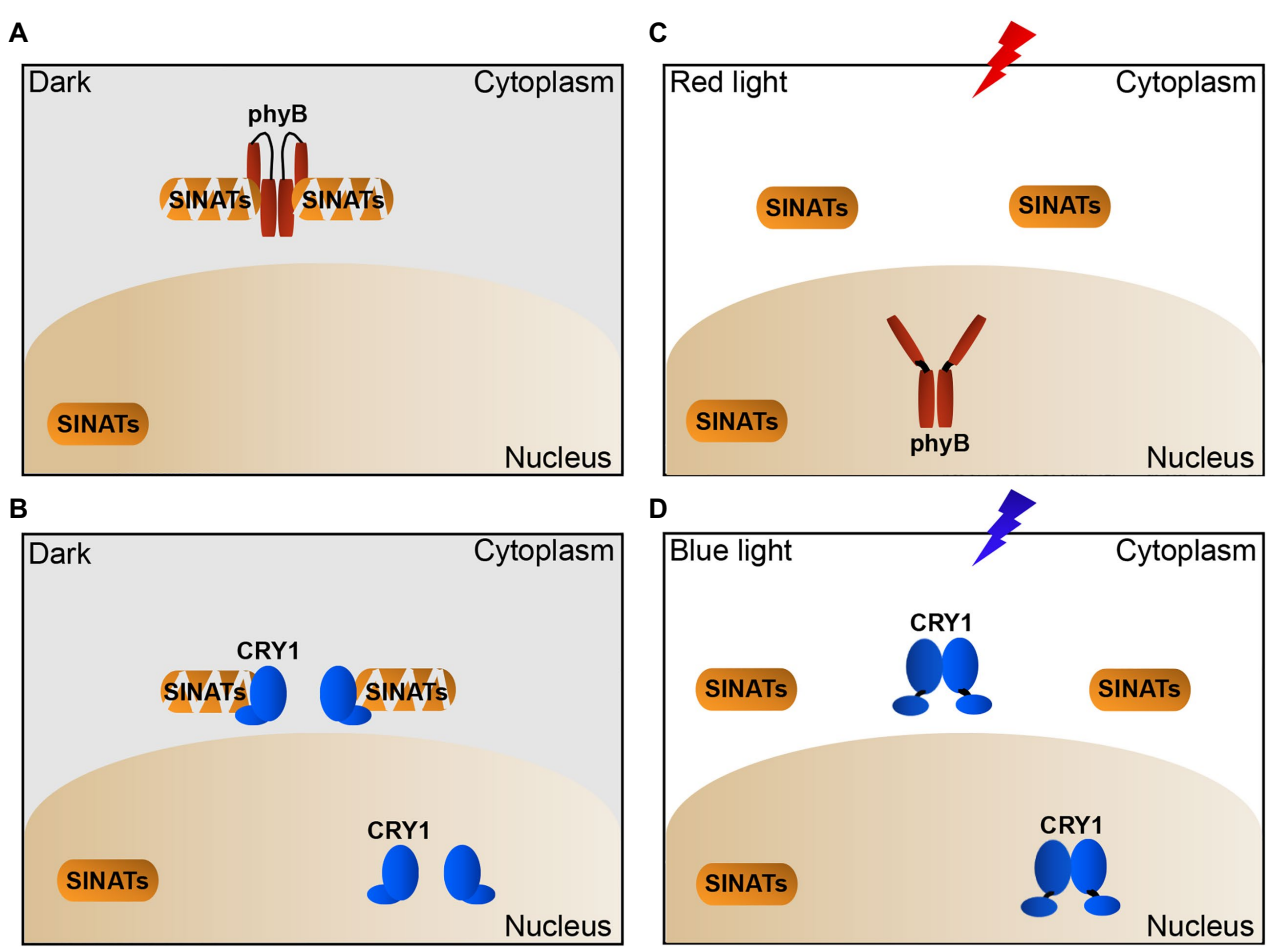
Proposed model illustrating how light-induced dynamic changes of phyB and CRY1 promote the accumulation of SINATs in cytoplasm. **(A,B)** In the dark, phyB and CRY1 in cytoplasm interact with SINATs to promote their autocatalytic ubiquitination and degradation. **(C)** In red light, phyB undergoes conformational change and translocates to the nucleus and dissociates from the cytosolic SINATs, which leads to SINAT accumulation in the cytoplasm. **(D)** In blue light, CRY1 in cytoplasm is activated and lead to its dissociation from the cytosolic SINATs and the accumulation of SINATs in the cytoplasm.

On the basis of previous studies and the results of this study, we propose a model for how phyB and CRY1 regulate SINAT protein levels in *Arabidopsis*. In the dark, phyB/CRY1 in the cytoplasm interacts with SINATs to promote their auto-degradation (Figures 5A,B). In red light, phyB translocates to the nucleus and blue light induces CRY1 to dissociate from SINATs, which facilitates SINAT protein accumulation (Figures 5C,D). This model well explained why light promotes the SINAT protein accumulation while the photoreceptors phyB and CRY1 directly promote the SINAT protein degradation. These findings provide insight into the light regulation of the protein stability of an E3 ligase through an association-dissociation mechanism that involves dynamic changes of photoreceptors.

## Data Availability Statement

The original contributions presented in the study are included in the article/Supplementary Material; further inquiries can be directed to the corresponding author.

## Author Contributions

XW and MY conceived the project. JH and YH conducted most of the experiments with the assistance of MY and XH. XW and JH wrote the manuscript. All authors contributed to the article and approved the submitted version.

## Conflict of Interest

The authors declare that the research was conducted in the absence of any commercial or financial relationships that could be construed as a potential conflict of interest.

## Publisher’s Note

All claims expressed in this article are solely those of the authors and do not necessarily represent those of their affiliated organizations, or those of the publisher, the editors and the reviewers. Any product that may be evaluated in this article, or claim that may be made by its manufacturer, is not guaranteed or endorsed by the publisher.

## Footnotes

^1^ http://imagej.nih.gov/ij/

## Abbreviations

SINATs: SINA of *Arabidopsis thaliana*
BR: brassinosteroid
phyB: phytochrome B
CRY1: cryptochrome 1
CHX: cycloheximide
COP1: Constitutive Photomorphogenic 1
