# Supplementary material for: Light-Induced Dynamic Change of Phytochrome B and Cryptochrome 1 Stabilizes SINATs in Arabidopsis
Source: Front Plant Sci. 2021 Aug 20;12:722733. doi: 10.3389/fpls.2021.722733 (PMC8417825; doi:10.3389/fpls.2021.722733)
Supplement: Supplementary file 1 [file Data_Sheet_1.docx]

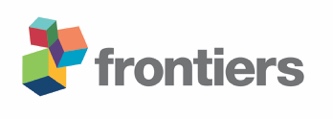


Supplemental Information


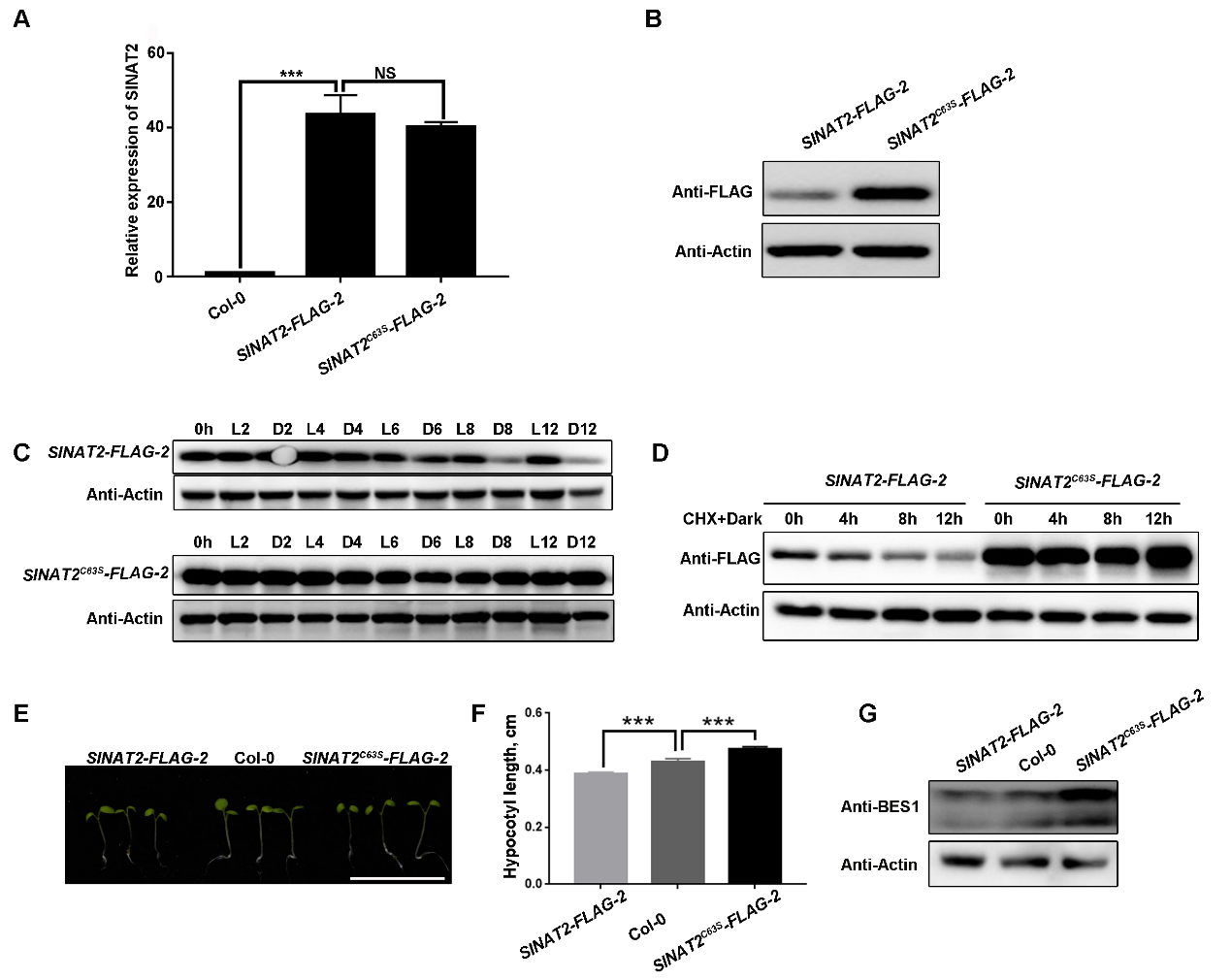


**Supplementary Figure 1. The degradation of SINATs in the dark is self-regulated.**

(**A**) Expression levels of the SINAT2 in Col-0, *SINAT2-FLAG-2* and *SINAT2*^C63S^*-FLAG-2* transgenic lines. Data are means ± SD (n = 3) and *P* values were determined by Student’s t-test; ****P* < 0.001; non-significant (NS), *P* > 0.05. (**B**) Protein level of SINAT2 in *SINAT2-FLAG-2* and *SINAT2*^C63S^*-FLAG-2* transgenic lines. (**C**) SINAT2 protein level under different light conditions in *SINAT2-FLAG-2* and *SINAT2*^C63S^*-FLAG-2* transgenic lines. (**D**) The half-life of SINAT2 protein in *SINAT2-FLAG-2* and *SINAT2*^C63S^*-FLAG-2* transgenic lines. (**E**) Hypocotyl phenotype of *SINAT2-FLAG-2*, Col-0, *SINAT2*^C63S^*-FLAG-2* transgenic lines. (**F**) Hypocotyl length of *SINAT2-FLAG-2*, Col-0, *SINAT2*^C63S^*-FLAG-2* transgenic lines. Error bars indicate SE (n=30). (**G**) BES1 protein level in *SINAT2-FLAG-2*, Col-0, *SINAT2*^C63S^*-FLAG* -2 transgenic lines.


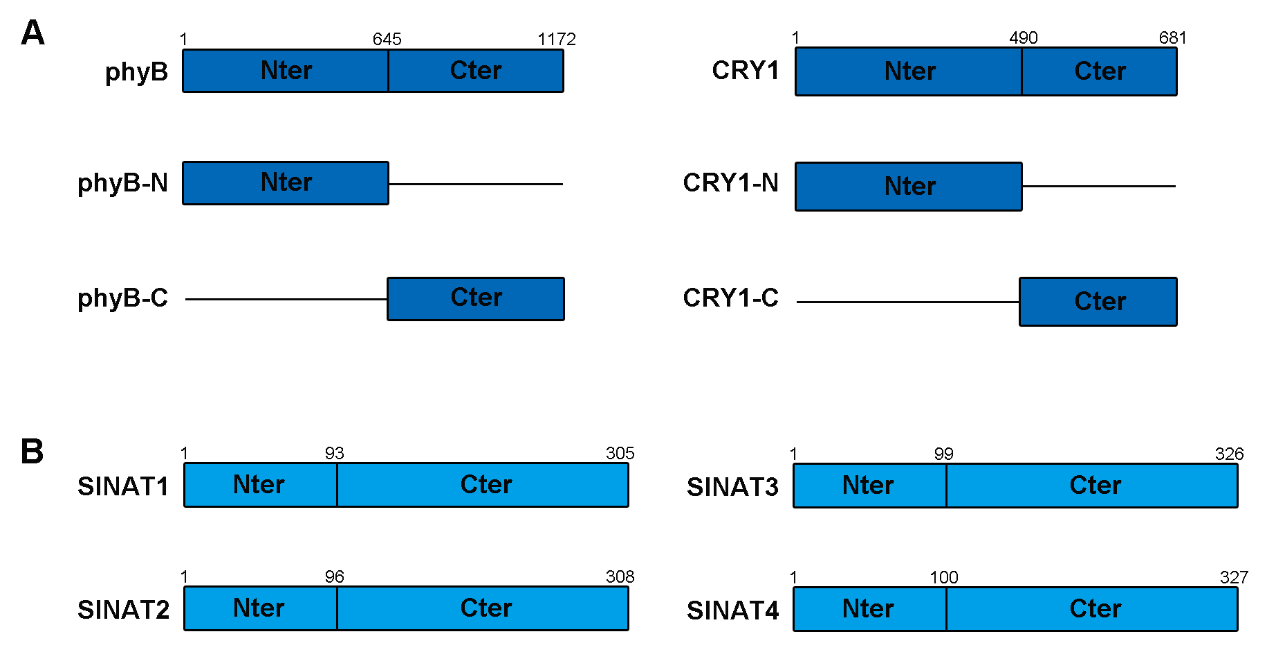


**Supplementary Figure 2. Truncated phyB and CRY1 variants.**

(**A**) Schematic diagram of full-length phyB, phyB-N (N-terminus of phyB), phyB-C (C-terminus of phyB), full-length CRY1, CRY1-N (N-terminus of CRY1), and CRY1-C (C-terminus of CRY1). (**B**) Schematic diagram of the N and C termini of the SINATs.


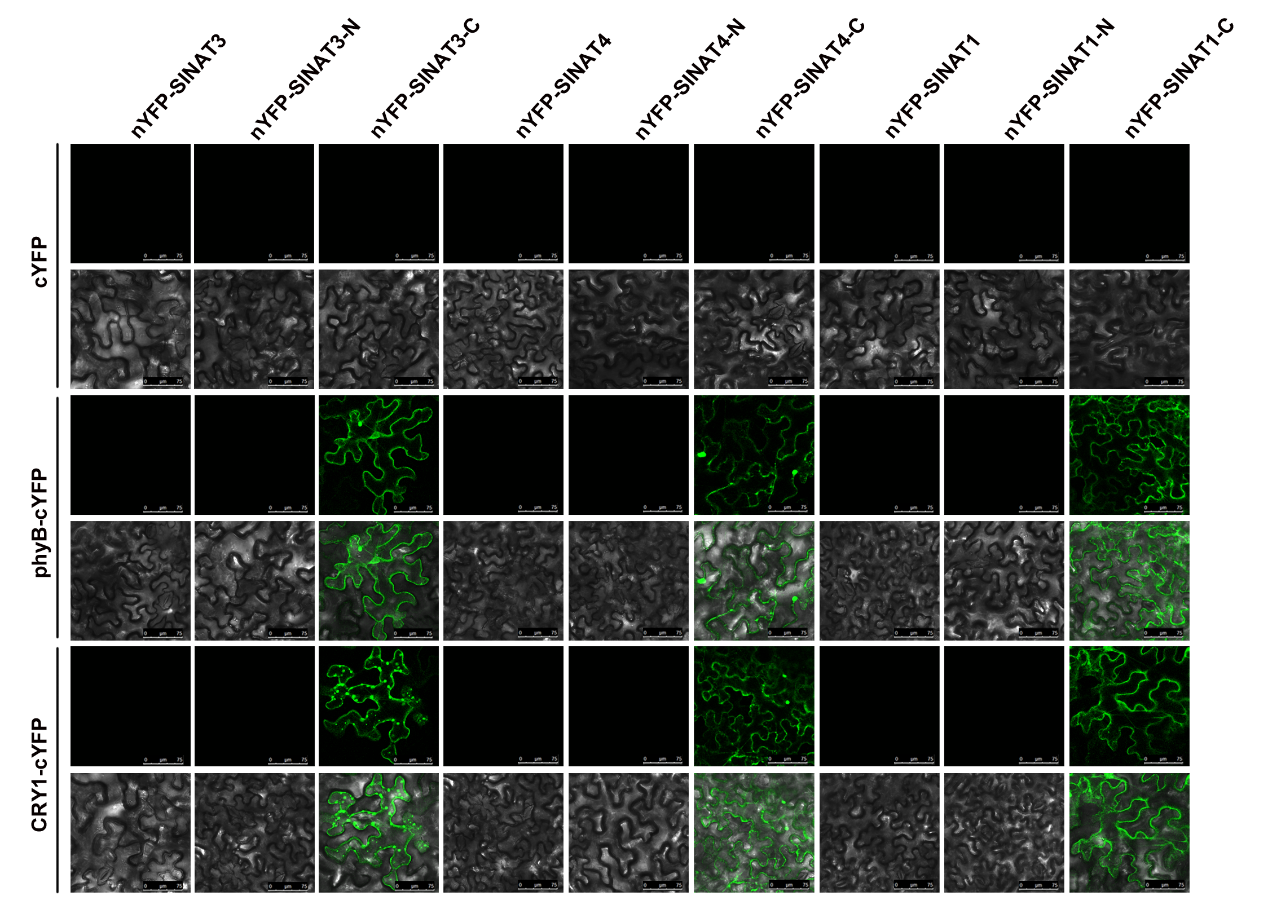


**Supplementary Figure 3. Interactions between SINAT1-C, SINAT3-C or SINAT4-C and phyB or CRY1 detected by BiFC assays.**





**Supplementary Figure 4. Confirmation of the genetic materials.**

(**A**) An anti-phyB antibody was used to detect phyB protein levels. (**B**) An anti-phyB antibody was used to detect phyB protein levels in *SINAT2-FLAG-2* and *SINAT2*^C63S^*-FLAG*.


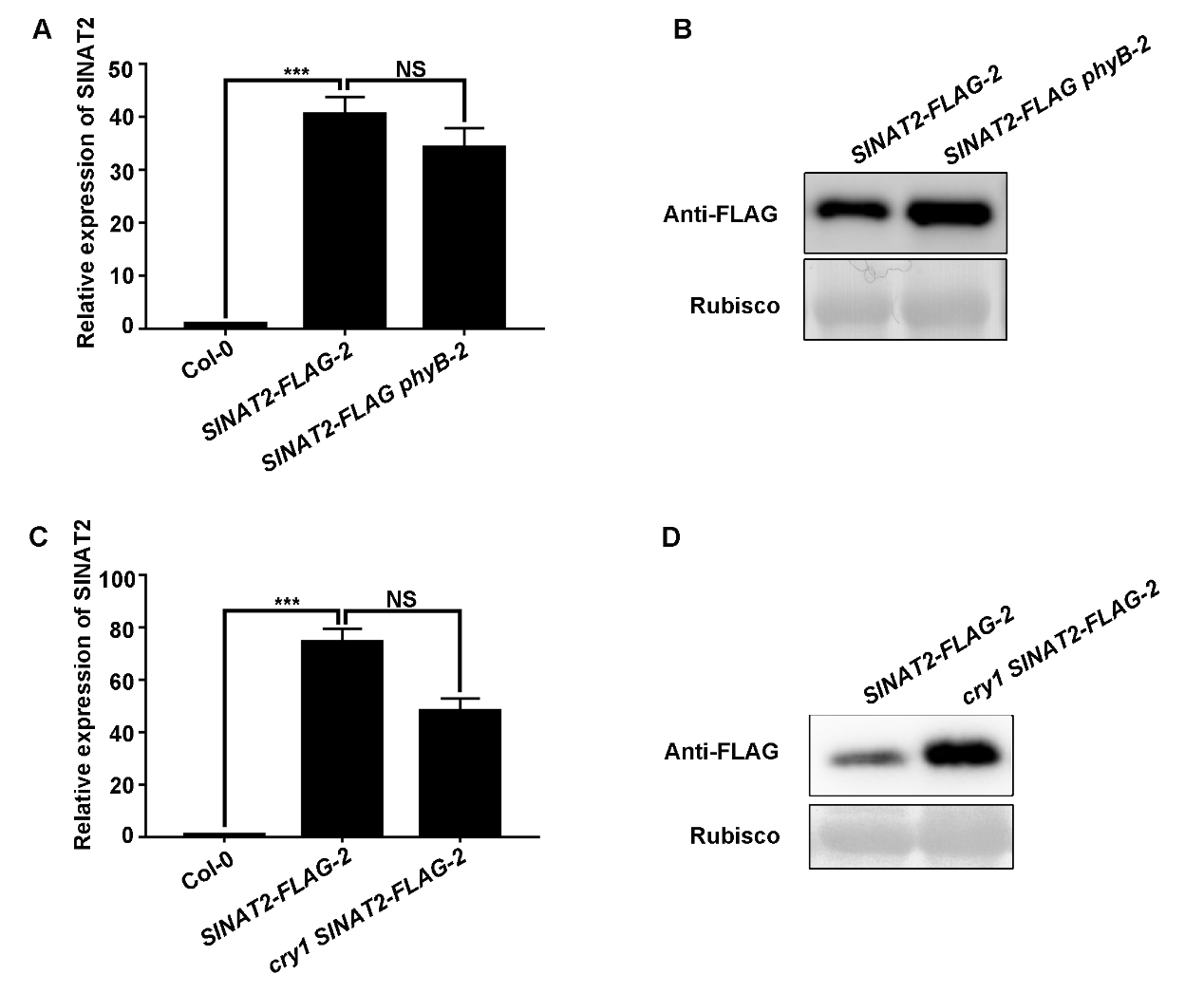


**Supplementary Figure 5. phyB and CRY1 promote the degradation of SINATs.**

(**A**) RNA expression levels of the SINAT2 in Col-0, *SINAT2-FLAG-2* and *SINAT2-FLAG phyB-2* hybrid line. Data are means ± SD (n = 3) and *P* values were determined by Student’s t-test; ****P* < 0.001; non-significant (NS), *P* > 0.05. (**B**) Protein level of SINAT2 in *SINAT2-FLAG-2* adult plants and *SINAT2-FLAG phyB-2* hybrid line. The amount of rubisco was used to quantify the total protein. (**C**) RNA expression levels of the SINAT2 in Col-0, *SINAT2-FLAG-2* and *cry1 SINAT2-FLAG-2* hybrid line. Data are means ± SD (n = 3) and *P* values were determined by Student’s t-test; ****P* < 0.001; non-significant (NS), *P* > 0.05. (**D**) Protein level of SINAT2 in *SINAT2-FLAG-2* adult plants and *cry1 SINAT2-FLAG-2* hybrid line.

**
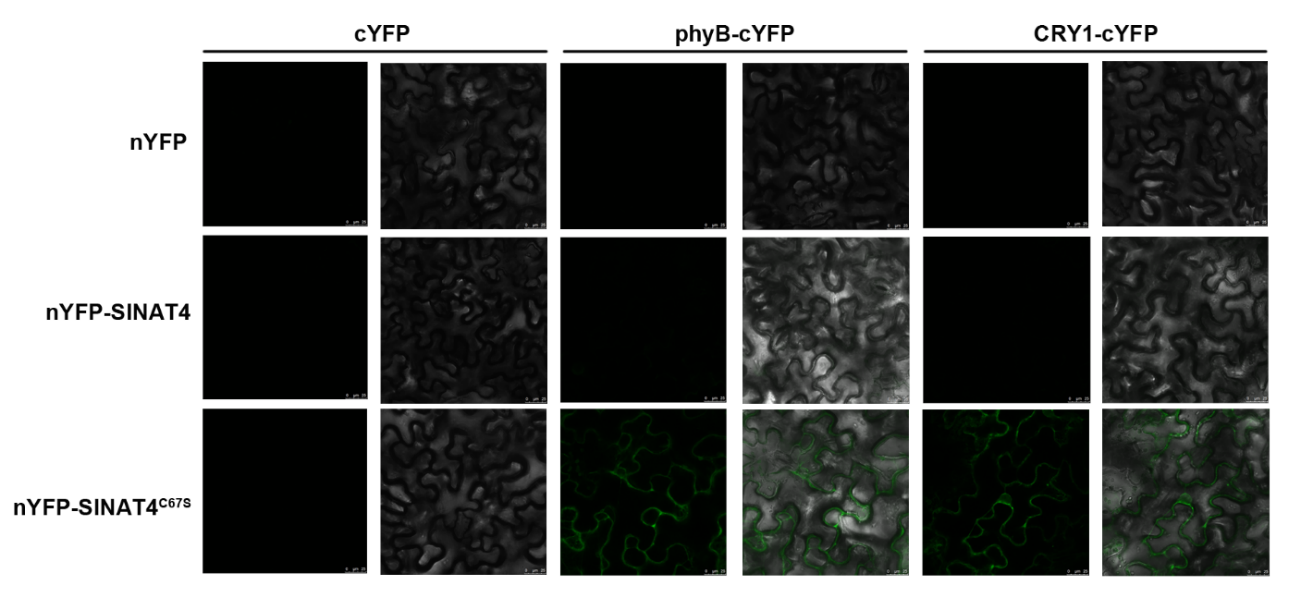
**

**Supplementary Figure 6. Interactions between SINAT4^C67S^ and phyB or CRY1 detected by BiFC assays.**

**
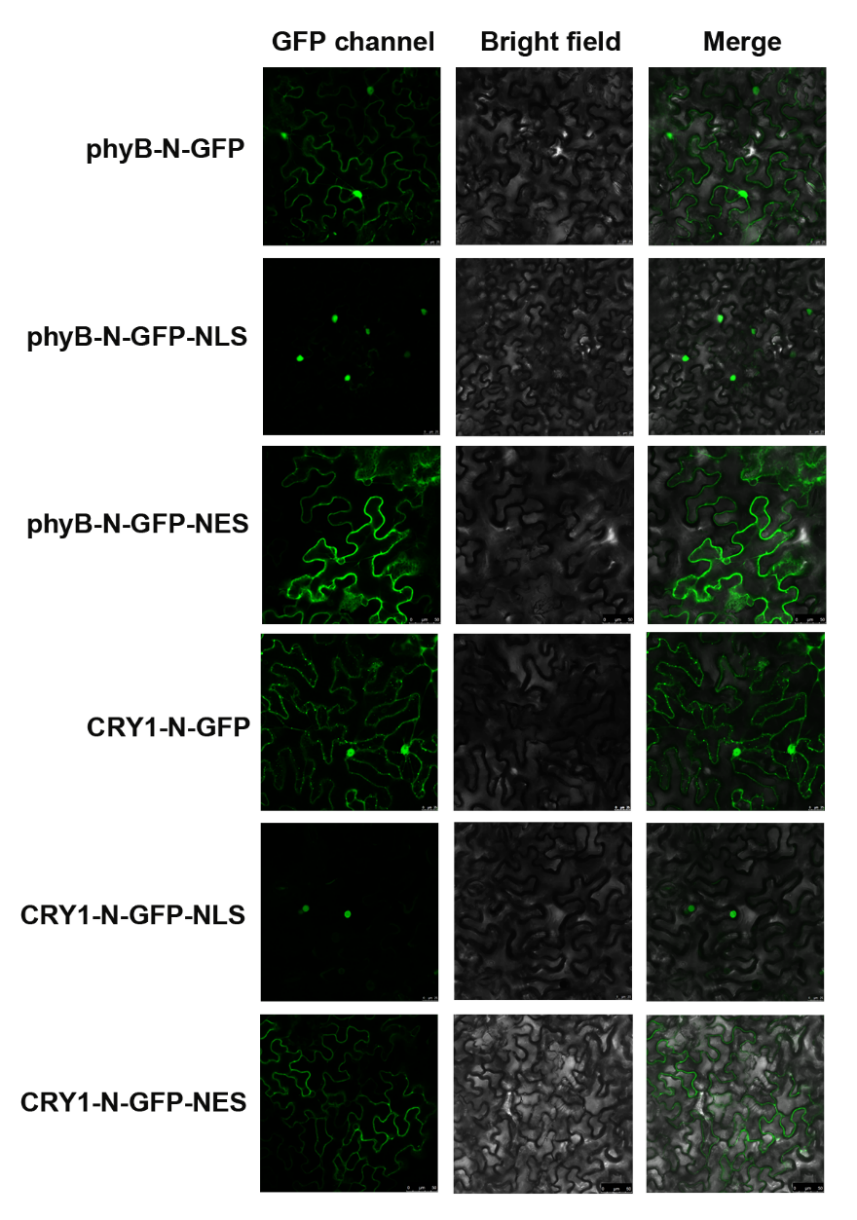
**

**Supplementary Figure 7. Subcellular localization of the indicated phyB variants.**

**
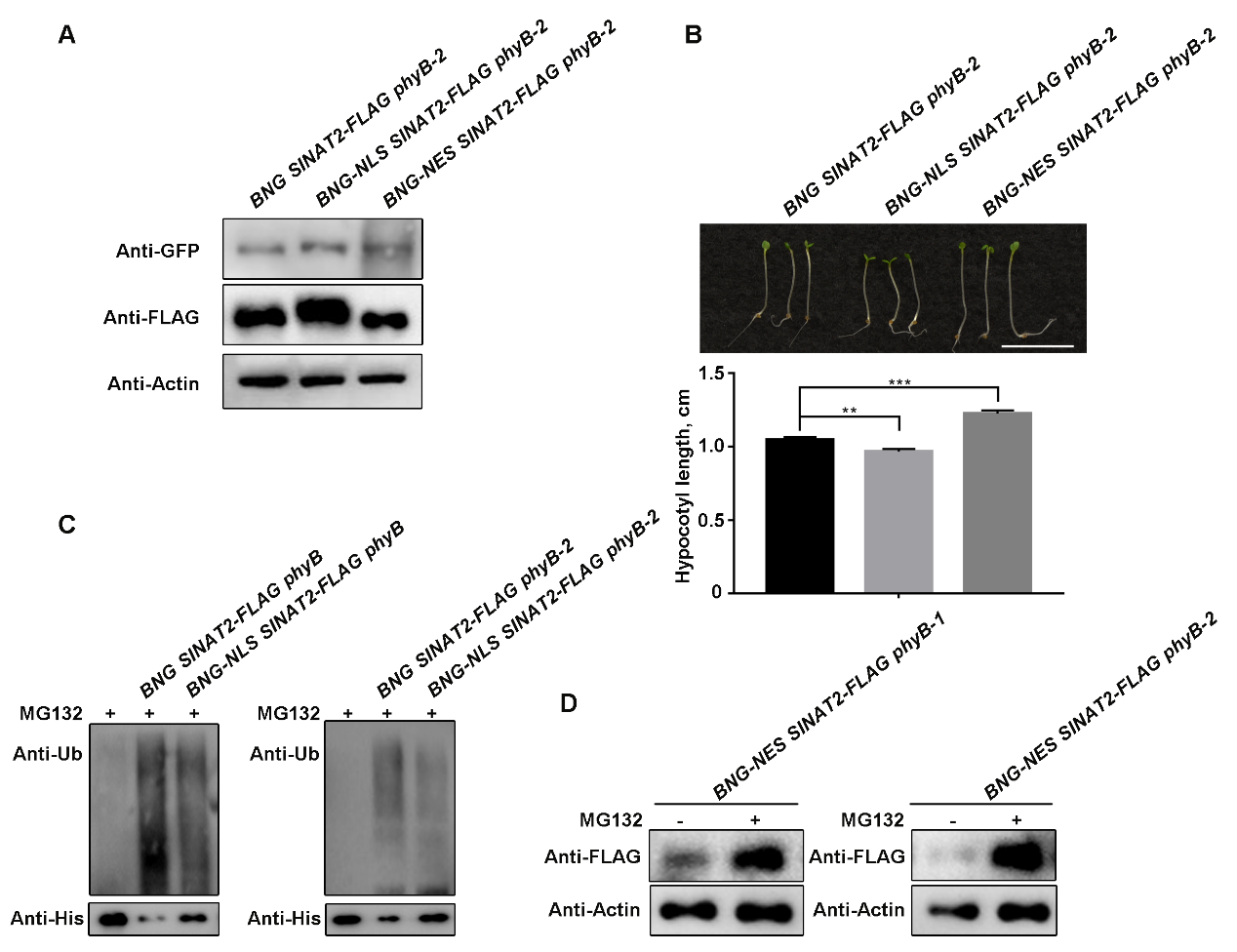
**

**Supplementary Figure 8. Subcellular localization of the indicated phyB variants.**

(**A**) SINAT2 protein levels in *BNG* *SINAT2-FLAG phyB-2, BNG-NLS SINAT2-FLAG phyB-2,* and *BNG-NES SINAT2-FLAG phyB-2* transgenic plants. An anti-GFP antibody was used to detect the phyB GFP fusion proteins. An anti-FLAG antibody was used to detect SINAT2-FLAG. (**B**) Hypocotyl phenotype and length of *BNG* *SINAT2-FLAG phyB-2, BNG-NLS SINAT2-FLAG phyB-2,* and *BNG-NES SINAT2-FLAG phyB-2* lines. Error bars indicate SE (n=30). Statistical significance was determined by Student’s *t*-test. **, *P* < 0.01. ***, *P* < 0.001. (**C**) Detection of His-SIANT2 ubiquitination in the *BNG* *SINAT2-FLAG phyB, BNG-NLS SINAT2-FLAG phyB* plants. (**D**) Detection of SINAT2 protein in *BNG-NES SINAT2-FLAG phyB* with or without MG132.
